# Graduates’ satisfaction with and attitudes towards a master programme in dental public health

**DOI:** 10.1186/s12909-015-0345-y

**Published:** 2015-03-26

**Authors:** Jaskiran Kahlon, Elsa Karina Delgado-Angulo, Eduardo Bernabé

**Affiliations:** 1 Division of Population and Patient Health, King’s College London Dental Institute at Guy’s, King’s College and St Thomas Hospitals, London, UK; 2 Departamento de Odontología Social, Facultad de Estomatología Roberto Beltrán Neira, Universidad Peruana Cayetano Heredia, Lima, Perú

**Keywords:** Dental public health, Postgraduate programme, Satisfaction, Attitudes

## Abstract

**Background:**

Monitoring graduates’ views of their learning experiences is important to ensure programme standards and further improvement. This study evaluated graduates’ satisfaction with and attitudes towards a Master programme in Dental Public Health.

**Methods:**

An online questionnaire was sent to individuals who completed successfully the Master of Science programme in Dental Public Health at King’s College London Dental Institute and had a valid email address. Participants provided information on demographic characteristics, satisfaction with and attitudes towards the programme. Satisfaction and attitudes scores were compared by demographic characteristics using multiple linear regression models.

**Results:**

Satisfaction scores with the programme were high, with 92% of respondents reporting the programme had met or exceeded their expectations. Learning resources and quality of teaching and learning were the aspects of the programme graduates were most satisfied with. The main motivations for taking the programme were to progress in career path and improve employment prospects. As for attitudes, 70.7% of respondents would recommend this course to a colleague or a friend. There were no significant differences in satisfaction and attitude scores by graduates’ demographic background.

**Conclusion:**

Graduates were satisfied with most aspects of the programme and reported positive attitudes towards it. This study highlights the value of using graduates’ views for programme’s improvement and the need for a regular monitoring of the programme.

## Background

It is generally agreed that any training programme must be evaluated for quality assurance and further improvement [1]. According to Kirkpatrick’s model of evaluation, there are four increasing levels to assess the impact of training programmes. Level one (*reaction*) measures how the person feels about the course; level two (*learning*) measures the extent to which principles, facts and techniques have been understood and absorbed; level three (*behaviour*) measures the application of the principles and techniques acquired on the job; and level four (*results*) measures the ends, goals and results desired [2,3]. Monitoring students’ reaction to their learning experiences is an activity that higher education institutions are increasingly undertaking [4-6]. This initial level of evaluation should be an inherent feature of every training programme because it offers ways in which a training programme can be enhanced and further developed. Also, it builds the base for higher levels of evaluation since reactions serve as a pointer as to whether learning is possible [1,3]. Students’ satisfaction with and attitudes towards training programmes are the most common indicators used to assess reaction [1,5]. However, there is additional value in exploring graduates’ reaction to training programmes because they are less emotionally attached to the institution and are back into work where they can judge whether the knowledge and skills acquired during the programme match their jobs requirements and responsibilities.

Satisfaction refers to how students’ experiences are met with their expectations [4,7]. On the other hand, attitudes are a mixture of beliefs, thoughts and feelings that predispose graduates to respond in a positive or negative way to institutions [8,9]. In addition to their role in ensuring learning and teaching quality standards, the two indicators serve as guidance for students, to aid decision-making at programme/institution level and to compute institutional performance indicators [10,11]. Higher education institutions are becoming increasingly interested in measuring students’ and graduates’ (customer’s) reactions because they promote internal restructuring, enhance their image, drive attention to students’ expectations and needs, provide data which will assist students’ performance in the labour market, and operate as a bridge with other disciplines [5]. Both indicators have been measured in relation to public health programmes in the past [12-17], but little has been documented on satisfaction and attitudes of graduates of postgraduate programmes in dental public health.

The Master of Science in Dental Public Health of King’s College London Dental Institute (KCLDI) aims to produce a highly knowledgeable individual capable and skilful in dental public health. It was established in the eighties to support the emerging new specialty of Dental Public Health in the United Kingdom (UK). The programme seeks to develop an understanding of the basic concepts of dental public health; the major health problems (and their determinants) of a community; the organisation of oral health services; research methods including epidemiology and statistics; and approaches to promoting oral health and preventing oral diseases. Students need to complete 180 credits over one year full-time or two years part-time. There are of seven core modules in the programme, which change from time to time according to staff expertise and emerging trends in the field. The current six taught modules are introduction to dental public health; principles of epidemiology; research methods; social and behavioural sciences as applied to medicine and dentistry; oral health promotion and education; and planning and evaluation of oral health, each counting for 20 credits. There is also a research module of 60 credits for which students need to complete and submit an original piece of research work for their dissertation. Practical experience is gained across some dental public health competencies by participating in various dental public health placements including teaching, research, health services and health promotion activities [18]. The aim of this study was to evaluate graduates’ satisfaction with and attitudes towards KCLDI Master Programme in Dental Public Health. This survey is a first step to address an important dimension of programme quality and the impact of educational programmes on public health dentists. The survey represents part of an ongoing, continuous quality improvement process being applied to KCLDI Master Programme in Dental Public Health.

## Methods

### Participants

A total of 141 students have graduated from the in-house Master of Science programme in Dental Public Health at KCLDI since 1981. The inclusion criteria for the study were students *(i)* who completed the programme successfully between 1981 and 2012 (those with at least 2 years of experience after graduation from the programme), *(ii)* who had a valid email address (where the recruitment email could be sent) and *(iii)* who agreed to participate voluntarily in the survey.

The study protocol was approved by KCL Biomedical Sciences, Dentistry, Medicine and Natural & Mathematical Sciences Research Ethics Subcommittee (Reference BDM/12/13-62). Return of the completed questionnaire was taken as implied consent.

### Data collection

A list of graduates, with their contact details, was not available at KCLDI and one had to be created for the study. We therefore proceeded to create group spaces in relevant social network sites (Facebook and Linked-in) through which we could advertise the survey and regain contact with graduates. These social media spaces were developed and run by the programme administrator, who acted as the gatekeeper during the data collection period.

Graduates who expressed interest in the survey were sent a recruitment email containing the details of the study, an invitation to participate and a link to the online questionnaire. The gatekeeper also asked participating graduates whether they could put her in contact with fellow graduates they knew would be interested in participating in the survey (i.e. snowball sampling). A reminder was emailed to all participants a week before the end of the survey period.

The questionnaire was developed based on previous relevant studies [12-17], the annual Postgraduate Taught Experience Survey (PTES) in the UK [6], and the programme specification forms [18]. An initial draft was circulated among past and present teaching and administrative staff involved in the programme for assessment. After amendments, the questionnaire was piloted with 5 graduates (i.e. past students who were either working or in further education at KCLDI) for validity assessment. Suggestions for improvements from the pilot study were incorporated in the final version of the questionnaire. The final format of the questionnaire was developed using the internet-based tool SurveyMonkey®. The questionnaire collected information on graduates’ background (sex, age, nationality and year of graduation), perception of skills gained during the programme, satisfaction with and attitudes toward the programme and professional development. This paper presents results on graduates’ satisfaction with and attitudes toward the programme only (level 1 in Kirkpatrick’s models of evaluation). Graduates’ satisfaction with the quality of teaching and learning, assessment and feedback, organisation and management, learning resources, skills and personal development, career and professional development and the overall experience of the programme was measured on semantic differential scales from −3 (when the course has definitely not met the graduate’s expectations) to 0 (when it has met the graduate’s expectations) to +3 (when the course has definitely exceeded the expectations). Two open-ended questions in this section asked graduates about the positive and negative aspects of the programme they would like to highlight. The final section of the questionnaire started with a list of topics to explore graduates’ motivations for joining the programme, and a follow-up question to ascertain the main motivation among those selected. Attitudes towards the programme were measured using a series of opinion statements on 5-point Likert scales ranging from 1 for ‘definitely disagree’ to 5 for ‘definitely agree’. Statements were related to a strong desire to take the course, suitability of the course to individual needs, work done during the course compared to others, wish to do a course at King’s regardless of the specialty, positive feelings towards dental public health as a result of the course, and value for money. Finally, graduates were asked whether they would recommend the programme to others and whether they would take the same decision of taking up the programme if given a chance today, which were answered using 5-point Likert scales ranging from 1 for ‘definitely not’ to 5 for ‘definitely yes’.

### Statistical analysis

All analyses were carried out in IBM® SPSS® Statistics version 20 for Windows. We first presented the distribution of satisfaction scores for the overall experience and each aspect of the programme using the mean, standard deviation (SD) and range of values. Scores were then compared by sex, age groups (<35, 35–44 and 45+ years), nationality (British, South Asian and Other) and time since graduation (before 2000, 2000–2010 and after 2010) using multiple linear regression. Bonferroni correction was used to reduce false positive results due to repeated comparisons over multiple outcomes (7 aspects of the programme) [19,20].

Thereafter, we presented the distribution of scores for the six attitude statements using the mean, SD and range of values. Attitude scores were then compared by background information (sex, age groups, nationality and time since graduation) using multiple linear regression. Bonferroni correction was used here as well to correct for multiple comparisons (6 attitudes) [19,20].

## Results

Fifty seven graduates met the inclusion criteria and 44 of them completed the online questionnaire (77% response rate). The characteristics of participants are shown in Table 1. Most participants were women (54.5%), younger than 35 years (45.4%) and from South Asian countries (45.4%). The average time since graduation was 8 years, with 38.6% of respondents graduated after 2010.

**Table 1:** **Characteristics of participants in the online survey**

Characteristic	Groups	n	(%)
Sex	Women	24	(54.6%)
	Men	20	(45.4%)
Age group	<35 years	20	(45.4%)
	35-44 years	13	(29.6%)
	45+ years	11	(25.0%)
Nationality	British	9	(20.5%)
	South Asian	20	(45.4%)
	Other	15	(34.1%)
Time since	After 2010	17	(38.6%)
Graduation	2000-2010	15	(34.1%)
	Before 2000	12	(27.3%)

The mean satisfaction score for the overall experience of the programme was 1.54 (SD: 1.21), with 92% of graduates reporting the programme had met or exceeded their expectations. Although there was variation in graduates’ satisfaction with different aspects of the programme, mean scores were all on the favourable side of the semantic scale or above the neutral point (Table 2). The aspects of the programme with the highest satisfaction scores were the learning resources (mean: 1.68; SD: 0.99) and quality of teaching and learning (mean: 1.66; SD: 1.22) whereas the aspect of the programme with the lowest satisfaction scores were assessment and feedback (mean: 0.93; SD: 1.52) (Table 2). There were no significant differences in the satisfaction scores by graduates’ sex, age group, nationality or time since graduation (multiple linear regression, p > 0.05 in all cases). Thirty and 24 graduates commented, respectively, on the positive and negative aspects of the programme in the open-ended questions. The most common positive aspects of the programme were the variety of instructors (n = 12), their expertise and enthusiasm (n = 10), working in small groups (n = 8) and opportunities to gain work experience with students’ placements (n = 7). The most frequent negative aspects of the programme were the focus just being restricted to the UK (n = 7) and the lack of proper career guidance (n = 6).

**Table 2:** **Graduates’ satisfaction scores with different aspects of the programme, by background characteristics**

Characteristics	Quality of teaching and learning		Assessment and feedback		Organization and management		Learning resources		Skills and personal development		Career and professional development		Overall experience	
	Mean	(SD)	Mean	(SD)	Mean	(SD)	Mean	(SD)	Mean	(SD)	Mean	(SD)	Mean	(SD)
*All participants*	1.66	(1.22)	0.93	(1.52)	1.20	(1.23)	1.68	(0.99)	1.41	(1.24)	1.49	(1.52)	1.54	(1.21)
*Sex*														
Women	1.36	(1.36)	0.64	(1.50)	1.00	(1.31)	1.45	(1.06)	1.00	(1.38)	1.14	(1.75)	1.14	(1.39)
Men	2.00	(0.94)	1.26	(1.52)	1.42	(1.12)	1.95	(0.85)	1.89	(0.88)	1.89	(1.10)	2.00	(0.75)
*p value* ^*a*^	*0.101*		*0.282*		*0.335*		*0.112*		*0.032* ^*b*^		*0.086*		*0.023* ^*b*^	
*Age group*														
<35 years	1.59	(1.54)	0.82	(1.67)	1.24	(1.25)	1.88	(1.17)	1.35	(1.50)	1.12	(1.93)	1.41	(1.42)
35-44 years	1.54	(1.05)	1.38	(1.19)	1.46	(1.05)	1.77	(0.93)	1.38	(1.26)	1.30	(1.18)	1.69	(1.11)
45+ years	1.91	(0.83)	0.55	(1.63)	0.82	(1.40)	1.27	(0.65)	1.55	(0.82)	2.27	(0.79)	1.55	(1.04)
*p value*^*a*^	*0.376*		*0.122*		*0.011* ^*b*^		*0.215*		*0.847*		*0.635*		*0.462*	
*Nationality*														
British	2.44	(0.53)	1.44	(1.33)	1.78	(0.67)	1.78	(0.83)	2.11	(0.78)	2.56	(0.53)	2.00	(0.71)
South Asian	1.39	(1.42)	0.94	(1.51)	1.17	(1.10)	1.72	(1.02)	1.22	(1.35)	0.72	(1.71)	1.28	(1.41)
Other	1.50	(1.09)	0.57	(1.65)	0.86	(1.56)	1.57	(1.09)	1.21	(1.25)	1.79	(1.19)	1.57	(1.16)
*p value*^*a*^	*0.469*		*0.841*		*0.454*		*0.771*		*0.269*		*0.249*		*0.699*	
*Time since graduation*														
After 2010	1.43	(1.43)	0.48	(1.69)	0.81	(1.40)	1.81	(1.12)	1.29	(1.42)	1.14	(1.77)	1.24	(1.37)
2000-2010	1.63	(0.92)	1.50	(1.20)	1.63	(0.74)	1.63	(0.74)	1.50	(1.07)	1.50	(1.31)	1.88	(0.99)
Before 2000	2.08	(0.90)	1.33	(1.23)	1.58	(1.00)	1.50	(0.90)	1.58	(1.08)	2.08	(1.00)	1.83	(0.94)
*p value*^*a*^	*0.232*		*0.055*		*0.025* ^*b*^		*0.579*		*0.936*		*0.210*		*0.135*	

^a^Groups were compared in multiple linear regression models for each satisfaction score.

^b^These p values are not significant. Significance level was set to (0.05/7=) 0.007 after Bonferroni correction.

**Figure 1:**
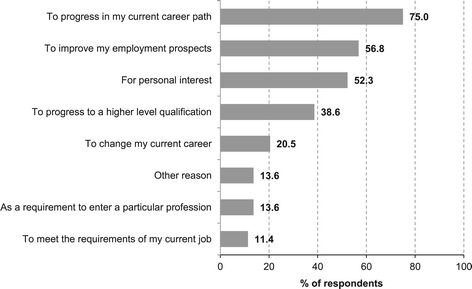
**Main motivations for taking up the programme.**

Figure 1 shows graduates’ motivations for taking the programme. To progress in current career path (75.0%), to improve employment perspectives (56.8%) and personal interest (52.3%) were the most common responses given by graduates. When asked to choose among those, graduates chose to progress in current career path as their main motivation (48.7%). As for attitudes towards the programme (Table 3), ‘as a result of this course, I have more positive feelings towards dental public health’ (mean: 4.24, SD: 1.04) and ‘I had a strong desire to do this course’ (mean: 4.17, SD: 0.97) were the statements with the highest scores while ‘the course was great value for money’ was the statement with the lowest score (mean 3.49, SD: 1.27). There were no differences in attitudes scores by graduates’ sex, age group, nationality or time since graduation (multiple linear regression, p > 0.05 in all cases). Finally, 70.7% of respondents would recommend this course to a colleague or a friend and 70.7% would still make the same decision to undertake the programme.

**Table 3:** **Graduates’ attitudes towards the programme, by background characteristics**

Characteristics	I had a strong desire to do this course		The course suited my individual needs perfectly		I worked harder on this course than on most courses		Wanted to take a course at King’s, regardless of the specialty		More positive feelings towards dental public health		The course was great value for money	
	Mean	(SD)	Mean	(SD)	Mean	(SD)	Mean	(SD)	Mean	(SD)	Mean	(SD)
*All participants*	4.17	(0.97)	3.68	(0.91)	3.88	(1.19)	2.83	(1.36)	4.24	(1.04)	3.49	(1.27)
*Sex*												
Women	4.27	(0.94)	3.59	(0.96)	3.91	(1.23)	2.68	(1.32)	4.14	(1.13)	3.23	(1.31)
Men	4.05	(1.03)	3.79	(0.85)	3.84	(1.17)	3.00	(1.41)	4.37	(0.96)	3.79	(1.18)
*p value*^*a*^	*0.513*		*0.673*		*0.840*		*0.466*		*0.453*		*0.110*	
*Age group*												
<35 years	4.18	(0.73)	3.53	(0.87)	4.00	(1.06)	3.00	(1.46)	4.35	(1.06)	3.53	(1.37)
35-44 years	4.08	(1.04)	3.85	(0.99)	3.92	(1.32)	3.31	(1.25)	4.38	(0.87)	3.15	(1.28)
45+ years	4.27	(1.27)	3.73	(0.90)	3.64	(1.29)	2.00	(1.00)	3.91	(1.22)	3.82	(1.08)
*p value*^*a*^	*0.763*		*0.813*		*0.801*		*0.098*		*0.669*		*0.296*	
*Nationality*												
British	4.22	(1.39)	3.89	(0.93)	4.00	(1.50)	2.11	(1.45)	4.00	(1.32)	4.11	(1.05)
South Asian	4.17	(0.86)	3.61	(0.98)	3.83	(1.34)	3.22	(1.40)	4.22	(1.11)	3.11	(1.23)
Other	4.14	(0.86)	3.64	(0.84)	3.86	(0.77)	2.79	(1.12)	4.43	(0.76)	3.57	(1.34)
*p value*^*a*^	*0.861*		*0.823*		*0.586*		*0.601*		*0.627*		*0.649*	
*Time since graduation*												
After 2010	4.33	(0.73)	3.81	(0.93)	4.14	(1.01)	3.00	(1.26)	4.38	(0.97)	3.52	(1.25)
2000-2010	3.75	(1.04)	3.25	(0.71)	3.50	(1.31)	3.00	(1.41)	4.25	(1.04)	2.75	(1.49)
Before 2010	4.17	(1.27)	3.75	(0.97)	3.67	(1.37)	2.42	(1.51)	4.00	(1.21)	3.92	(1.25)
*p value*^*a*^	*0.802*		*0.960*		*0.867*		*0.273*		*0.956*		*0.456*	

^a^Groups were compared in multiple linear regression models for each satisfaction score. Significance level was set to (0.05/6=) 0.008 after Bonferroni correction.

## Discussion

Graduates reported high levels of satisfaction with the overall experience of the programme, with 9 out of every 10 graduates stating the programme had met or exceeded their expectations. This finding is consistent with results from the PTES among current students, where overall satisfaction scores with the programme have remained high in the last few years. Taken together, they demonstrate continued satisfaction with the programme, even several years after completion. However, there is room for improvement in spite of the consistently high satisfaction across the six aspects of the programme. Assessment and feedback was the aspect of the programme graduates felt least satisfied with (even though 80.5% reported the programme had met or exceeded their expectations in this area). It is difficult to identify the troubling areas graduates referred to in this domain, mainly because assessment and feedback was not raised as an issue in the open-ended questions. It is possible that some graduates were not satisfied with their final grades. However, results from the PTES show feedback is the least satisfactory aspect of higher education in the UK, especially in relation to timeliness and helping clarify students’ understanding [6]. Tutors and students have different perceptions on what effective feedback means, with tutors often believing their feedback is more useful than students do [21-24]. In an internal focus group run immediately after the survey, students said that effective feedback means identifying the positive and negative aspects of their work, linking performance to marks and receiving criticism from tutors. They praised timely feedback and individual face-to-face dialogue with tutors. The importance of feedback on summative work (rather than exclusively on formative work) was also recognised by students for managing stress, coping with possible failure and lifelong learning. Since then, the programme and modules have been revised in line with those recommendations.

In the open-ended questions, graduates advocated for removing the exclusive focus on the UK healthcare system and providing proper career guidance. Starting from October 2012, the focus of the programme changed to cater for a large cadre of overseas students, where the British healthcare system is only a study case while some seminars and assignments are tailored to students’ countries of origin. The lack of career guidance is an important issue as graduates’ main motivations for taking the programme were to progress in current career path and to improve the employment prospects. Most students now see postgraduate training as an investment for strengthening career development and improving employability, rather than just taking up any programme at any institution [6]. Interestingly, this same group of graduates provided evidence that the programme has helped them with career progress in the short-term in before-and-after comparisons, as measured by the proportion moving to higher education institutions and taking up leadership/managerial roles in their organisations (Aslam S, Delgado-Angulo EK, Bernabé E: Perceived learned skills and professional development of graduates from a Master Programme in Dental Public Health, submitted). However, it would be beneficial for students to have opportunities (seminars, career days, etc.) where they can discuss with tutors and relevant counselling services their career prospects [12].

Graduates also reported favourable attitudes towards the programme, with 7 out of every 10 stating that they would recommend the programme to a friend or colleague. Graduates reported more positive feelings towards public health dentistry as a result of the course and a strong desire to take this course (as opposed to the low scores given to the premise of taking a course at King’s regardless of the specialty). These results are suggestive of graduates’ values or regard for public health dentistry in general and the master programme in particular. On the other hand, there was a feeling among some graduates that the course was not value for money. This result is quite understandable given the relatively high tuition fees that international students have to pay for education in the UK in addition to living arrangements of living in London for a year to complete the programme.

Some limitations of this study need to be addressed. First, participants were recruited using non-random sampling and the final sample size was relatively small. Although participation rate was high, we could not recruit participants from all calendar years (especially from older cohorts). Thus, the present results cannot be generalised to the full population of graduates and they may reflect short- rather than long-term outcomes of the programme. Second, this study assessed satisfaction with and attitudes towards the programme, the two of which refer to level one of Kirkpatrick’s framework only [2,3]. All four levels of the framework are interrelated and level one builds the base for higher levels of evaluation, for graduates’ views of the programme (reaction) serve as a pointer as to whether learning is possible [1,3]. Further research is needed to assess other levels of Kirkpatrick’s framework though. Third, the survey was based on graduates’ self-reports, which are prone to overestimation and measurement bias. Responses could be influenced by graduates’ emotional commitment to their alma mater and gratitude for receiving a professional degree [25,26]. However, students are a critical source of programme evaluation [4,7-9]. We gave participants chances to voice negative aspects of the programme and some took that opportunity, thus increasing the credibility of our findings.

## Conclusion

Graduates from the Master of Science in Dental Public Health of King’s College London were very satisfied with most aspects of the programme, although they advocated for certain improvements. Graduates also reported positive attitudes towards the programme, with 7 out of every 10 graduates stating they would recommend the programme to a colleague or a friend.
